# Retention and performance of peer educators and sustainability of HIV prevention services for adolescents in the Zimbabwe Smart-LyncAges project: an ecological study

**DOI:** 10.11604/pamj.2022.41.131.29539

**Published:** 2022-02-16

**Authors:** Simbarashe Mabaya, Ronald Ncube, Hannock Tweya, Collins Timire, Jeffrey Karl Edwards, Wole Ameyan, Nonhlahla Zwangobani, Talent Makoni, Aveneni Mangombe, Sinokuthemba Xaba, Julia Samuelson

**Affiliations:** 1 World Health Organization, Harare, Zimbabwe,; 2 The International Union Against Tuberculosis and Lung Disease, Harare, Zimbabwe,; 3 The Lighthouse Trust, Lilongwe, Malawi,; 4 The International Union Against Tuberculosis and Lung Disease, Paris, France,; 5 University of Washington, Department of Global Health, Seattle, Washington, USA,; 6 World Health Organization, Geneva, Switzerland,; 7 Zimbabwe National Family Planning Council, Harare, Zimbabwe,; 8 Ministry of Health and Child Care, Harare, Zimbabwe

**Keywords:** Smart-LyncAges, adolescent, Zimbabwe

## Abstract

**Introduction:**

in 2016, the partner-funded Smart-LyncAges participatory learning project explored the feasibility of a youth-friendly package including incentivized peer educators (PEs) to enhance adolescent sexual and reproductive health (ASRH) and voluntary medical male circumcision (VMMC) linkages. After 12 months of implementation, funding reduction resulted in reduced direct project monitoring and discontinuation of monetary incentives for PEs. We assessed if reduced funding after one year of implementation affected the performance and retention of PEs and uptake of VMMC and HIV testing in ASRH services by adolescents in Bulawayo City (urban) and Mount (Mt) Darwin District (rural) in Zimbabwe.

**Methods:**

our study was an ecological study using routine data collected from March 2016 to February 2017 (intensive support) and March 2017 to February 2018 (reduced support). All the ASRH and VMMC sites in Mt Darwin and Bulawayo were involved. Participants included 58 PEs and all adolescents accessing VMMC and ASRH services. Retention of PEs measured by the submission of monthly reports and uptake of VMMC and HIV testing were the primary outcome measures.

**Results:**

the Smart-LyncAges project engaged 58 PEs with 80% aged 20-24 years. Two-thirds were male and 60% were engaged in peer education before the project. Retention of PEs was not negatively affected by funding reduction, with 70% retained up to 11 months after funding reduction. However, their performance, measured by submission of monthly activity reports and the number of adolescents reached with VMMC and HIV messages, declined while uptake of both VMMC and HIV testing was sustained.

**Conclusion:**

sustained uptake of services was possibly due to heightened awareness of service availability and demand generation in the first year of implementation. Peer-led interventions are effective for health information dissemination. Monetary incentives determine performance, but are not the only reason for retention.

## Introduction

In East and Southern Africa, Human Immunodeficiency Virus (HIV) is the most important cause of years of life lost among adolescent boys aged 10-19 years [1]. In 2007, World Health Organization (WHO) and the Joint United Nations Program on HIV/AIDS (UNAIDS) recommended that in countries with a high HIV prevalence, voluntary medical male circumcision (VMMC) should be part of a comprehensive HIV prevention package and an entry point for additional sexual and reproductive health (SRH) services for this sub group [2]. However, there is evidence that adolescents often face significant barriers in accessing HIV prevention services. These challenges include unfriendly health services due to negative attitudes of service providers and the community about their sexuality [3-5]. The legislative environment in many countries prevents sexually active adolescents from independently seeking HIV testing services, an important entry point to comprehensive HIV prevention and other SRH services [5]. In addition, a systematic review of adolescent sexual reproductive health (ASRH) services in the context of provision of VMMC revealed that in most countries health services addressing the specific needs of male adolescents were generally not available, a threat to the benefits of VMMC programming for this important sub population [5]. In Zimbabwe, adolescents remain disproportionately affected by HIV despite a sustained decline in HIV incidence rates among those aged 15-49 years. In 2017, it was estimated that 12.4% of all new HIV infections occurred in adolescents from 10-19 years old [6]. Since 2006, Zimbabwe has been implementing an ASRH program targeting young people aged 10-24 years.

The ASRH package addresses underlying risk factors for HIV acquisition, such as unsafe sexual practices, alcohol and substance abuse with significant overlap with the VMMC package which was launched in 2009. The target for the VMMC program is to reach 80% of men and boys (1.3 million) at highest risk of HIV infection (10-29 years) [7]. By 2018, Zimbabwe had cumulatively circumcised 1.3 million males [8]. Integration of VMMC and ASRH services was reported to improve health outcomes of adolescents [5,9], however, there was dearth of literature from program settings [5]. In March 2016, the Ministry of Health and Child Care (MOHCC) in Zimbabwe, with support from WHO, implemented the Smart-LyncAges project, to explore ways of sustaining and enhancing adolescent services including through linkages between VMMC and ASRH services in Bulawayo City (urban) and Mt Darwin district (rural). In March 2017, the funding for the project was reduced with the hope that sites would continue implementing activities perceived as important to ensure sustainability of the intervention benefits. With reduced funding, direct supervision of the peer educators was reduced with only remote follow up, and their monthly allowances were stopped. Little attention has been given to the sustainability of the intervention, that is the continued implementation of the activities after reduced funding in the project sites. We therefore set out to assess if the reduction in funding support to the Smart-LyncAges project from February 2017 to March 2018 affected; i) the retention and performance of peer educators after discontinuation of monthly allowance (retention was defined as being recorded as active in the peer educators´ registers at the youth centers and attending activities at the same centers and performance by the submission rates of monthly reports, and trends in the numbers they reached with VMMC and HIV messages; ii) uptake of both VMMC and HIV testing in ASRH services among adolescent boys (10-19 years).

## Methods

**Study design:** this was an ecological study utilizing previously collected routine data.

**Study populations:** the study focussed on all peer educators who were working in Smart-LyncAges project in Bulawayo and Mt Darwin between March 2016 and February 2018. In addition, it focused on all adolescent boys (10-19 years) who were circumcised and those who were tested for HIV in ASRH services in the two locations during the same time period were included.

### Setting

**General setting:** Zimbabwe has a population of over 13,5 million inhabitants with the majority 68%, residing in rural areas [10]. Mt Darwin is a predominantly Rural district and one of seven districts in Mashonaland Central Province with a population of 212,725, the 15-19 years age group accounting for 11% of the population [11]. Bulawayo is the second-largest city in Zimbabwe, with a population of 653,337, the 15-19 years age group accounting for 12% of the population [11]. Both Mt Darwin and Bulawayo are priority hotspots, with medium to high risk factors for HIV transmission and HIV prevalence of 11.5% and 19.8% among those aged 15-49 years in 2014 respectively [12].

**Voluntary medical male circumcision program:** voluntary medical male circumcision services in Bulawayo have been offered in a vertical approach. There were two dedicated VMMC sites, namely Bulawayo male circumcision center in the city center and the Lobengula male circumcision site in the western suburb's area where the Smart-LyncAges project was implemented. Both sites provide static and outreach services. In Mt Darwin, VMMC services were more integrated into the general health services, being offered primarily at the District Hospital, with a few outreach sites such as Dotito Rural Hospital. The VMMC package includes HIV testing services, sexually transmitted infections (STIs) screening and treatment. Condoms are also provided at no cost to the clients.

**Additional sexual and reproductive health program:** additional sexual and reproductive health services in Mt Darwin have been offered through two youth centers that offer education and entertainment as well as clinical services to young people aged 10-24 years. Clinical services offered at youth centres include HIV testing, STIs screening and treatment and provision of contraception. Bulawayo has 15 youth centers that offer education and entertainment and no clinical services. In Bulawayo, clinical ASRH services are provided at two Zimbabwe National Family Planning Council (ZNFPC) clinics. In the two locations ASRH services were provided at no cost to the young people. As part of the ASRH package, comprehensive sexuality education has been given in schools, with peer education also a key component of the ASRH program. Peer educators have been volunteers aged between 10 and 24 years who were expected to be role models from their communities. Their roles include providing information to their peers in groups or as individuals and referring them to appropriate services, motivating and supporting positive behavior change, and distributing information, education, and communication (IEC) materials and condoms. Prior to the Smart-LyncAges project, peer educators were supposed to receive a monthly stipend of US$20, but this has been discontinued for more than a year due to funding challenges.

**The Smart-LyncAges project:** consultative work on the participatory learning Smart-LyncAges Project started in 2014 with national and local stakeholders, with implementation commencing in March 2016. Implementation started with sensitization of the additional different stakeholders in the two project areas including the health authorities, young people, parents, and different organizations that worked with young people. Capacity building of service providers and peer educators with subsequent refresher trainings were conducted with a focus on the provision of VMMC information and youth friendly services. A service directory was developed showing the locations and contact details of all organizations providing services targeted at young people in each area. Referral slips were developed, which were used mainly by peer educators to refer clients between ASRH and VMMC services. Information, education, and communication materials with joint VMMC and ASRH messages were developed and distributed by peer educators and through the youth centers. The IEC materials developed included posters and fliers, which were also distributed through social media. Social media platforms used included WhatsApp groups for peer educators and service providers and a Facebook page. U- report, an interactive short message service (SMS) platform developed by the United Nations International Children's Emergency Fund (UNICEF) was used to disseminate uniform as well as conduct opinion polls on various topics. The project used the routine systems that were already in use for reporting and monitoring in the VMMC and ASRH programs. This included ZNFPC and population services international (PSI) supervising and monitoring ASRH and VMMC activities respectively, with MOHCC providing oversight. A dedicated coordinator was recruited who conducted monthly visits to the sites and organized quarterly stakeholder project review meetings. A monthly allowance of US$15 was paid to each peer educators upon submission of a monthly report. After the project implementation had started, additional peer educators were recruited, while some stopped participation at different times throughout the duration of the project. Each youth center received a monthly allowance of US$20 for airtime to facilitate communication and a quarterly seed fund of US$100 to support minor renovations and improvements. This active support was provided for the period March 2016 to February 2017. The funding support was subsequently reduced between March 2017 and February 2018 to only cover salary support for the project coordinator with reduced supervision.

**Data variables, sources of data and data collection:** anonymized demographic data on peer educators were abstracted from youth center registers and monthly reports into a structured pro forma and entered into EpiData software v2.2.2.182, (EpiData Association, Odense, Denmark). Each youth center keeps a register with demographic characteristics showing the dates when they were engaged and when they discontinued peer education. The registers also record when the monthly reports are received. All the peer educators who were active before the start of Smart-LyncAges project were taken as having started peer education in March 2016 and all those who were active at the end of the study period were taken to having stopped peer education in February 2018. Aggregated data on VMMC service uptake were obtained from the national reporting system (District Health Information Software 2 (DHIS2)) and those on HIV testing uptake from youth center and ZNFPC clinic registers. Data on numbers reached by peer educators with VMMC and HIV messages were abstracted from the monthly reports submitted by the peer educators. Peer educators´ performance measured by the proportions of expected reports that were submitted comparing the two-time periods was analyzed for trends. The expected number of reports was equal to the number of peer educators who were registered at the youth centers as being active during that month, and the submitted reports were the actual number that submitted their reports. Retention of peer educators was also analyzed for the two time periods. Continuous variables e.g. age were summarized using medians and interquartile ranges (IQRs). Categorical variables (education, sex) were summarized using frequencies and proportions of clients who were either tested for HIV or were circumcised during VMMC were stratified by reporting year (intense project support or reduced project funding).

**Ethical approval:** permission for the study was obtained from MOHCC (ASRH and VMMC programs). Ethics approval was obtained from the local Medical Research Council of Zimbabwe (MRCZ) and The Union Ethics Advisory Group in Paris, France. The research did not collect primary data and adolescents were not involved in the design, or conduct, or reporting, or dissemination of our research study.

## Results

**Demographic characteristics of peer educator:** the Smart-LyncAges project engaged 58 peer educators in Bulawayo and Mt Darwin between March 2016 and February 2018. Table 1 shows their demographic characteristics. Four in five were aged between 20 and 24 years. Two-thirds were male and the majority, (60%) had been recruited before the Smart-LyncAges project. More than half of the peer educators (55%) were unemployed. The minimum age was 17 years and the maximum 24 years.

**Table 1:** demographic characteristics of peer educators in the Smart-LyncAges project in Mt Darwin District and Bulawayo City March 2016-February 2018

Variable	N	(%)
**Total**	58	(100)
**Age category in years**		
15-19	12	(20.7)
20-24	46	(79.3)
Median (Interquartile range)	21	(20-22)
**Sex**		
Male	38	(65.5)
Female	20	(34.5)
**Employment status**		
Unemployed	32	(55.2)
Self employed	7	(12.1)
Employed	19	(32.7)
**Education**		
Secondary level	48	(82.8)
Tertiary level	10	(17.2)
**Timing of recruitment as peer educator**		
Before Smart -LyncAges project	35	(60.3)
During Smart-LyncAges project	23	(39.7)

**Demographic characteristics of clients who accessed VMMC and ASRH services:** Table 2 and Table 3 show the demographic characteristics of the clients that accessed VMMC and ASRH services in Bulawayo and Mt Darwin from March 2016 and February 2018.

**Table 2:** age distribution of clients circumcised in Mt Darwin District and Bulawayo City, March 2016-February 2018

	Year 1 - period of intensive project support (March 2016 - February 2017)		Year 2-period of reduced project support (March 2017 to February 2018)	
	N	(%)✝	N	(%)✝
Total	15 183	100	14 823	100
**Age groups in years**				
Oct-14	3198	21.1	6538	44.1
15-19	3901	25.7	3534	23.8
20+	8084	53.2	4751	32.1

✝ column percentages

**Table 3:** age distribution of clients tested for HIV in ASRH services in Mt Darwin District and Bulawayo City, March 2016-February 2018

	Year 1-period of intensive project support (March 2016 - February 2017)		Year 2-period of reduced project support (March 2017 to February 2018)		p-value
	N	(%)✝	N	(%)✝	
**Total**	4273	100	3994	100	
**Age groups in years**					
10-14	290	6.8	767	19.2	
15-19	1641	38.4	1216	30.4	<0.001
20+	2342	54.8	2011	50.4	

**Figure 1:**
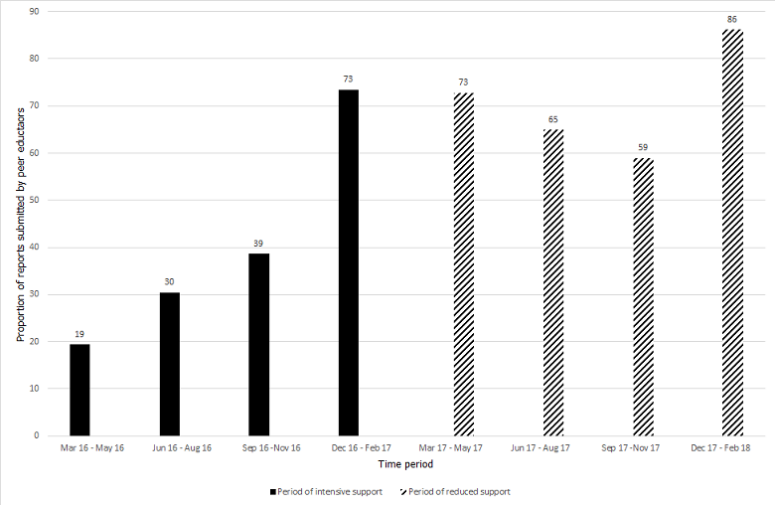
proportion of expected reports submitted by peer educators in the Smart-LyncAges project in Mt Darwin District and Bulawayo City, March 2016 February 2018

**Figure 2:**
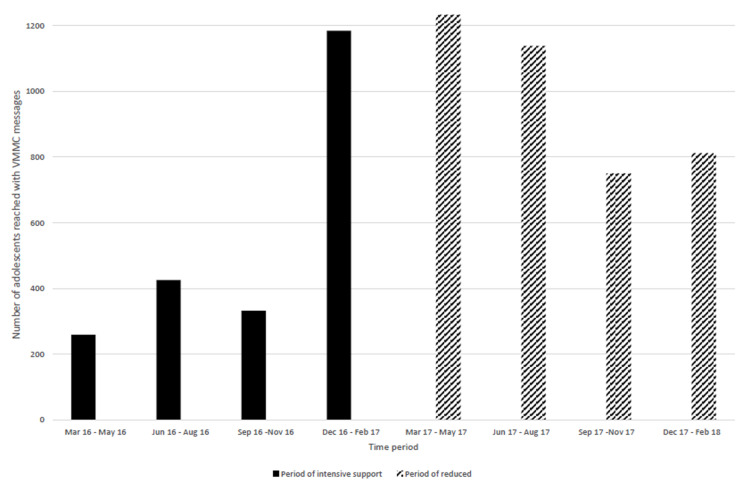
trends in adolescents reached with VMMC messages during the Smart-LyncAges project in Mt Darwin District and Bulawayo City, March 2016-February 2018

**Figure 3:**
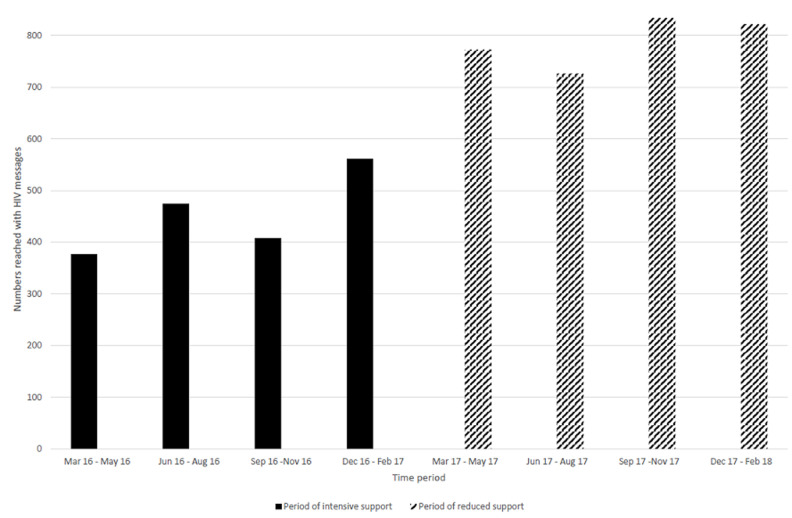
trends in adolescents reached with HIV messages among adolescents during the Smart-LyncAges project in Mt Darwin District and Bulawayo City, March 2016-February 2018

**Performance of peer educators:** during the period of intensive project support (March 2016 - February 2017) the proportion of reports submitted by peer educators increased from 19% in the first quarter (March 2016 - May 2016) to 74% in the fourth quarter (December 2016 - February 2017). Submission of reports however declined thereafter during the period when project support was reduced (March 2017- February 2018), though remaining above levels when the project started, with the lowest submission rate of 59% between September 2017 and November 2017 (92 submitted out of 156 expected reports). Notably, the last quarter of reduced project support had the highest reporting rate of 86% (Figure 1). A total 38 peer educators were actively engaged when the project started in March 2016 support while 43 were actively engaged when the reduced project support period started (March 2017). During both time periods, the peer educators worked for an average of 11 months (SD 2.4 and 0.8 respectively). Trends in adolescents reached with VMMC messages by peer educators are shown in Figure 2. The number of adolescents reached with VMMC messages increased during the time of intensive project support, from 259 in the first quarter (March 2016 - May 2016) to 1185 in the fourth quarter (December 2016 - February 2017). The number reached thereafter declined to 750 between September 2017 and November 2017. The number of adolescents reached with HIV messages by peer educators continued on an upward trend despite the reduction in project support to a peak in the quarter September 2017 to December 2017 (Figure 3).

**Figure 4:**
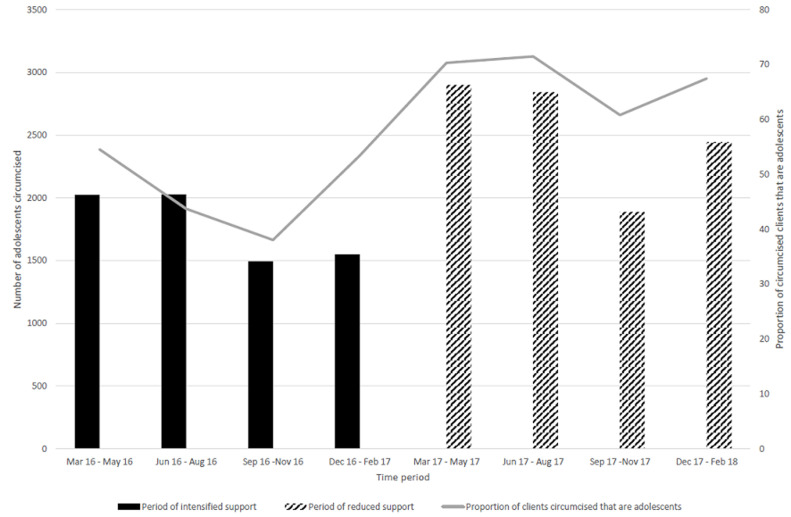
trends in the numbers and proportions of circumcised adolescents during the Smart-LyncAges project in Mt Darwin District and Bulawayo City March 2016-February 2018

**Figure 5:**
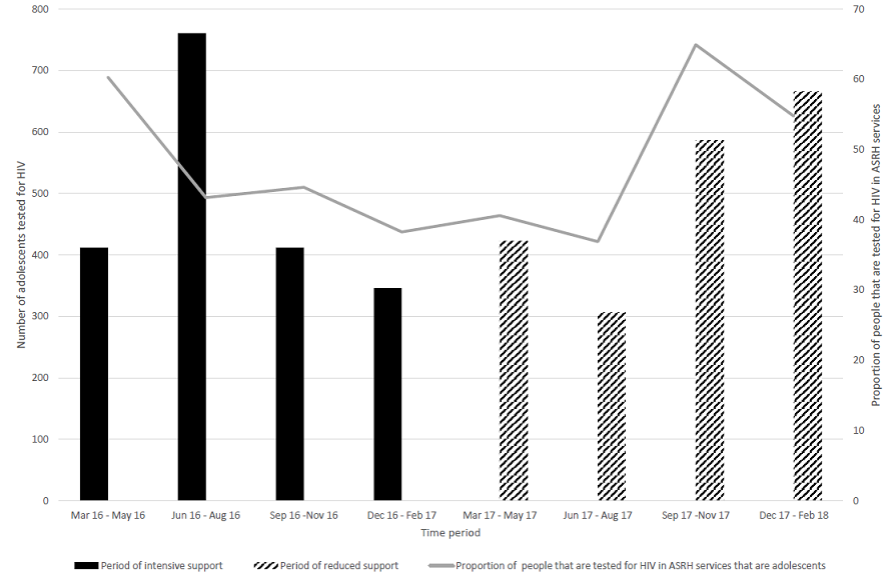
trends in the numbers and proportion of adolescents tested for HIV during the Smart-LyncAges project in Mt Darwin District and Bulawayo City March 2016-February 2018

**Uptake of VMMC and HIV testing services among adolescents:** the proportions of circumcised clients who were adolescents and the proportions of adolescents testing for HIV in ASRH services increased gradually even during the period of reduced support (Figure 4, Figure 5). Therefore, the proportion of adolescents among circumcised clients and those tested for HIV was greater when support was reduced than during the intensive project support (p<0.001) (Figure 4, Figure 5).

## Discussion

Our study attempts to answer a pertinent concern of most funders of innovative public health interventions, who invariably want to know if their investment leads to residual beneficial outcomes, or such outcomes fade away after seed funding is spent or substantially reduced [13]. To our knowledge this retrospective record review is the first in Zimbabwe to explicitly document programmatic sustainability within the context of the partner funded Smart-LyncAges project. While we observed reduction in the level of performance by peer educations, almost all peer educators worked for the whole period of the reduced support and uptake of VMMC and HIV testing services continued to increase when funding was reduced. Despite reduced project funding, peer educators continued work for almost the entire period of the project (average retention time was 11 months). Possibly, these peer educators were influenced by intrinsic motivation as reported in other settings [14]. This could have been the case in our setting as some peer educators were already active before the Smart-LyncAges project. Retention has also been reported to be influenced by other non-monetary benefits derived from participation in similar adolescent peer education programs [15]. It is plausible that the observed retention was related to non-monetary incentives in addition to ASRH services, such as vocational and arts training provided especially at the youth centers in Bulawayo.

The performance of peer educators as measured by submission of activity reports and trends in adolescents reached with VMMC messages however declined over time when funding was reduced. Monetary incentives in peer education programs have been documented to influence performance. Through peer education is voluntary, there is an associated opportunity cost with giving time to programs and incentives are often key enablers in ensuring commitment [16]. This is consistent with our findings that despite high retention, commitment to project activities seemed to be reduced with the removal of monetary incentives. Incidentally, there was a notable spike in expected reports submitted by peer educators in the last quarter of reduced support. Around this time, there were discussions to resume intensive support with the peer educators' incentive. This could possibly explain the spike in reporting as peer educators anticipated the incentives. It implies that the training and intensive supervision increased their competency and likely confidence. Studies elsewhere have shown that peer education programs often thrive with intensive monitoring and support of activities, an assertion shared by a systematic review that peer educators require close supervision to deliver [17]. When funding was reduced there was reduced supervision and monitoring of peer educators by the project coordinator, and that could explain the decline in performance with reduced funding support. Most of the peer educators in our study were “older” young people (ages 20-24 years), which could have been related to recruitment bias, which tends to focus on out of school young people in our settings. A systematic review by Medley *et al*. showed that peer educators communicate more effectively with peers of similar demographic characteristics or risky behavior, which may have contributed to declining trends in the number of adolescents reached by predominantly older (non-adolescent) peer educators with VMMC messages [16].

Among other major barriers to accessing ASRH services among adolescents reported in Zimbabwe and Kenya was a lack of knowledge of where services were located and related costs of accessing such services [3]. In addition, myths and misconceptions about the VMMC procedure has been noted a barrier to accessing the service by adolescents in Zimbabwe [17]. The Smart-LyncAges project leveraged on the network of peer educators and a service directory, as well as social media platforms to disseminate such information and dispel prevalent myths and misconceptions about VMMC and HIV to intended beneficiaries. It is possible that in the second year of reduced support, the intended beneficiaries had been made more aware during the first year of the locations of services, including myths and misconceptions related to VMMC and HIV and that the services were for free. This could have contributed to the observed sustained uptake of services after a reduction in project funding. In addition, possible confounded may have occurred by non-project related national VMMC and HIV testing services (HTS) scale-up initiatives. The attitude of service providers has been extensively described as a deterrent to the uptake of ASRH services, including VMMC and HIV testing, among adolescents [18]. One of the key components of the Smart-LyncAges service package was the capacity building of service providers on the provision of youth-friendly services. It is also plausible that the improved attitude of service providers could have contributed to the sustained increase in service uptake beyond the intensive project support.

**Strengths and limitations:** the study adhered to the Strengthening the Reporting of Observational Studies in Epidemiology (STROBE) guidelines on the conduct of observational studies, and portrayed typical programme settings; it can therefore be used to inform decision making and possible intervention scale up [19]. However, the study had some limitations. First, an obvious limitation inherent to such observational studies is the possibility of data documentation errors that could not be validated. Second, aggregated data were used to explore uptake of VMMC and HIV testing services which did not allow tracking individuals adolescents who were reached with messages to receiving VMMC and/or HIV testing services. Lastly an ecological analysis has serious limitation regarding other factors that are effecting outcomes.

## Conclusion

This study has some important implications. First, while peer education is a viable approach in disseminating key public health messages to adolescents, sustaining performance of peer educators is vulnerable to continued provision of various types of support including incentives. There is need to explore to what extent non-monetary incentives and intensive supervision could be leveraged to enhance performance. Second, the high retention of peer educators despite reduced support needs further exploration on factors associated with retention to better inform possible scale up of more sustainable peer led interventions. This may include the value of PE as an initial step in a job trajectory. Lastly, sustained uptake of health services by adolescents requires strong health systems that ensure that services are available and easily accessible. In conclusion, continued investment in innovative public health approaches is critical, however for peer led interventions, monetary incentives were not the only determinant of peer educator retention and sustainable uptake of adolescent health services.

### What is known about this topic

- It is already known that peer education can increase knowledge levels among adolescents.

### What this study adds

- This study adds new knowledge that monetary incentives may be important to incentivize peer educators to perform but they are not the only factor; peer education can therefore be a sustainable way of providing health information even when donor funding is reduced.
